# Rituximab in Systemic Lupus Erythematosus: Transient Effects on Autoimmunity Associated Lymphocyte Phenotypes and Implications for Immunogenicity

**DOI:** 10.3389/fimmu.2022.826152

**Published:** 2022-04-08

**Authors:** Francesca Faustini, Natalie Sippl, Ragnhild Stålesen, Karine Chemin, Nicky Dunn, Anna Fogdell-Hahn, Iva Gunnarsson, Vivianne Malmström

**Affiliations:** ^1^ Division of Rheumatology, Department of Medicine, Karolinska Institutet, Karolinska University Hospital Solna, Stockholm, Sweden; ^2^ Center for Molecular Medicine, Karolinska Institutet, Stockholm, Sweden; ^3^ Department of Clinical Neuroscience, Karolinska Institutet, Stockholm, Sweden

**Keywords:** systemic lupus erythematosus, rituximab, double negative B-cells, age-/autoimmunity-associated B-cells, T follicular helper cells, T peripheral helper cells, PD-1, immunogenicity

## Abstract

B cell abnormalities are common in systemic lupus erythematosus (SLE), and include expansion of double negative (DN) and age-associated-like B cells (ABC-like). We aimed to investigate rituximab (RTX) effects on DN and ABC-like B-cell subsets and, when possible, also secondary effects on T cells. Fifteen SLE patients, fulfilling the ACR 1982 criteria, starting RTX and followed longitudinally up to two years, were analyzed for B- and T- lymphocyte subsets using multicolor flow cytometry. DN were defined as IgD-CD27- and ABC-like as CD11c+CD21- within the DN gate. Additional phenotyping was performed adding CXCR5 in the B-cell panel. Cellular changes were further analyzed in the context of the generation of anti-drug antibodies (ADA) against RTX and clinical information. The SLE patients were mainly females (86.6%), of median age 36.7 (29.8-49.4) years and disease duration of 6.1 (1.6-11.8) years. Within the DN subset, ABC-like (IgD-CD27-CD11c+CD21-) B cell frequency reduced from baseline median level of 20.4% to 11.3% (p=0.03), at early follow-up. The DN B cells were further subdivided based on CXCR5 expression. Significant shifts were observed at the early follow-up in the DN2 sub-cluster (CD11c+CXCR5-), which reduced significantly (-15.4 percentage points, p=0.02) and in the recently described DN3 (CD11c-CXCR5-) which increased (+13 percentage points, p=0.03). SLE patients treated with RTX are at high risk of developing ADA. In our cohort, the presence of ADA at 6 months was associated with lower frequencies of DN cells and to a more pronounced expansion of plasmablasts at early follow-up. The frequency of follicular helper T cells (T_FH_, CD4+PD-1+CXCR5+) and of peripheral helper T cells (T_PH_, CD4+PD-1+CXCR5-) did not change after RTX. A sub-cluster of PD-1^high^CD4+ T cells showed a significant decrease at later follow-up compared to early follow-up (p=0.0039). It is well appreciated that RTX transiently influences B cells. Here, we extend these observations to cell phenotypes which are believed to directly contribute to autoimmunity in SLE. We show early transient effects of RTX on ABC-like memory B cells, later effects on PD-1^high^ CD4+ cells, and possible implications for RTX immunogenicity. Further insight in such effects and their monitoring may be of clinical relevance.

## Introduction

Systemic lupus erythematosus (SLE) is an autoimmune disease in which a break of tolerance towards self leads to the onset of autoreactive immune responses, which in turn, are followed by the production of multiple autoantibodies with subsequent inflammation and tissue damage (1). SLE is characterized by multiple defects of the immune system, both innate and adaptive, with the involvement of several cell types, including B- and T-lymphocytes. Such alterations are both qualitative and quantitative, with a correlate of peripheral blood lymphopenia, which is a clinical hallmark of the disease (2, 3).

With regard to the B-cell compartment, profound aberrations have been described, which involve several functional and maturation stages. Within the typical lymphopenia of SLE patients, naïve B cells are majorly affected, while memory cells and plasmablasts are expanded, and correlate with disease duration and activity (3, 4). Among the memory B cells, a subpopulation lacking the surface marker CD27 has been described as enriched and also is significantly associated with clinical manifestations, activity and autoantibodies (5, 6). These cells are known as double negative B cells (DN, IgD-CD27-). Their discovery has paved the way for a more extensive study of the B-cell memory compartment in SLE, which, together with studies conducted in other settings (e.g. infections) has led to the definition of novel subsets collectively known as “atypical memory B-cells” (7). Recently, phenotypical and functional analysis unraveled that the expansion of DN B cells in SLE patients is attributable to a sub-phenotype denominated DN2, which lacks the homing receptor CXCR5 and the complement receptor CD21, but expresses the integrin subunit CD11c (8). These cells, which are characterized by a T-bet driven transcriptional programme, are otherwise described as ABCs (age-associated/autoimmunity-associated B cells) or ABC-like (moniker herein adopted) (9–11).

Murine ABCs and their human counterparts have received increasing attention over the last years. First described in mouse models as accumulating with age, and being largely represented in female strains prone to autoimmunity, they have also been investigated in humans (9–11). In the context of human immunology, these cells, besides their accumulation with age, have been demonstrated to expand in response to chronic antigenic stimulation, as during infectious diseases such as malaria and HIV (11). There is an ongoing discussion whether they are exhausted or post-activated cells or if they represent, in settings of chronic stimulation, a transitional stage towards antibody forming cells. In autoimmune diseases, ABC-like cells seem to accumulate prematurely, and to represent precursors of antibody forming cells with an enriched autoreactive BCR repertoire (9, 12–14). Moreover, ABC-like cells have been reported to correlate with disease activity in SLE and identified as active players in renal tissue in lupus nephritis (13, 15).

In T-dependent immune responses, essential for the B cells to evolve into antibody secreting cells is interaction with T cells, which takes place in lymphoid tissues. In germinal centres (GC), CD4+T follicular helper (T_FH_) provide help to B cells, promoting them to undergo molecular events such as somatic hypermutation, class switching, affinity maturation, and generation of memory cells and long-lived plasma cells (16). The phenotype and function of T_FH_ has in part been explored in SLE, where these cells have been found expanded in peripheral blood, and to correlate with disease manifestations such as lupus nephritis (LN) both in humans and in mouse models (17, 18). In the context of autoimmunity, T-B cell interactions occur even outside of the lymph nodes, namely in the inflamed tissues, *via* the formation of GC-like structures (19). The investigation of such interactions has led to the identification of a peripheral counterpart of T_FH_. So-called T peripheral helper cells (T_PH_) are PD-1^high^ CXCR5- CD4+ T cells and have been previously identified in the synovium of ACPA+ rheumatoid arthritis patients (20). In SLE, T_PH_ were recently found to be expanded in peripheral blood and associated with disease activity and CD11c+ B-cell frequency, suggesting their ability to drive B-cell responses on a functional level (21). This fits with the notion of ABC-like cells being precursors of antibody-producing cells as introduced above.

Rituximab (RTX), a B-cell depleting anti-CD20 antibody, is currently used in several autoimmune diseases, and as a rescue treatment for SLE patients, especially in the case of LN (22). CD20 is expressed on most B cells, starting from pre-B cells to memory B cells (23). Hereby, RTX does not have a direct effect on plasma cells and early B cell precursors, since they do not express CD20 (5, 23). Several studies have investigated the effect of RTX on B- and partly on T-cell subsets, however, the effect of RTX on the above described pathogenic subsets (ABC-like, T_FH_ and T_PH_ cells) has so far not been studied in the context of SLE. Thus, we examined the longitudinal effect of RTX on the frequency of the DN subsets, CD11c+CD21- ABC-like, T_FH_ and T_PH_ cells up to two years after RTX treatment. Moreover, we explored whether anti-drug antibodies (ADA) directed against RTX may show any relation with the residual cells during B-cell depletion.

## Material and Methods

### Patients

The study prospectively included 15 SLE patients who underwent treatment with original RTX (Mabthera ^®^). All patients fulfilled the SLE 1982 classification criteria (24).

The study was approved by the local ethics committee at Karolinska University Hospital and each patient gave written informed consent prior to inclusion.

Demographic and clinical data were collected from the electronic clinical charts of the patients. These include age, gender, disease duration at RTX start, disease activity measures using SLE Disease Activity Index 2000 (SLEDAI-2K) (25) and main indication for RTX treatment. Records were also revised to retrieve information about ongoing and previous treatments with antimalarials and immune suppressants (DMARDs). Data concerning laboratory markers such as total white blood cell and lymphocyte counts at baseline, anti-dsDNA antibody and complement activation status were also collected. These measurements were conducted at the clinical laboratories of the Karolinska University Hospital according to clinical routine.

Since the measurements of anti-dsDNA and complement fractions (C3 and C4) have been conducted with different methods over the years, we collected the relative information as categorical variables.

### Treatment

RTX was administered over the years according to two possible schedules: a hematological regimen (375 mg/m^2^ of body surface area (BSA) administered weekly over four weeks), generally in association with intravenous cyclophosphamide and/or corticosteroid pulses as described elsewhere (26), or according to a rheumatoid arthritis-like regimen (1000 mg two weeks apart), or a lighter version of it (500 mg two weeks apart) with or without corticosteroid pulse therapy according to the judgement of the treating physician.

### Lymphocyte Isolation and Flow Cytometry Analysis

Blood samples were collected at baseline and follow-up visits and peripheral blood mononuclear cells (PBMC) were isolated and cryopreserved following Ficoll density gradient centrifugation. For flow cytometry experiments, PBMC at baseline and follow up were thawed and stained at the same time together with one buffy coat from a healthy blood donor. Around 1 x 10^6^ PBMCs for each panel were labeled with LIVE DEAD Fixable NEAR IR Dead Cell Stain Kit (Invitrogen) and further stained with fluorescent antibodies for B, and T cell marker. For the B cell panel, cells were stained with anti-CD3, CD14, CD16, CD19, IgD, CD27, CD38, CD11c, CD21 and, when possible, anti-CXCR5 antibodies. For the T-cell panel, cells were labeled with anti-CD16, CD14, CD19 and CD3, CD4, CD8, PD-1, CCR7, CXCR5, CD45RA antibodies (**Supplementary Table S1**). B- and T-cell panel samples were run on the BD Biosciences FACS Verse Flow Cytometer. Data was processed using the FlowJo 10.7.1 software (FlowJo). The gating strategies are shown in **Supplementary Figure S1**.

Samples with less than 100 events in the CD19+ gates were excluded from the B-cell phenotyping.

The status of B-cell depletion (BCD) upon RTX treatment was defined as a CD19% value below 0.5.

### Anti-Drug Antibodies

The presence of ADA was determined with an in-house validated electrochemiluminescence bridging assay (ECL) according to a previously described protocol (27, 28).

### Statistical Analysis

Statistical analyses were performed using Prism 7 software (Graph Pad, San Diego, CA, USA).

**Figure 1:**
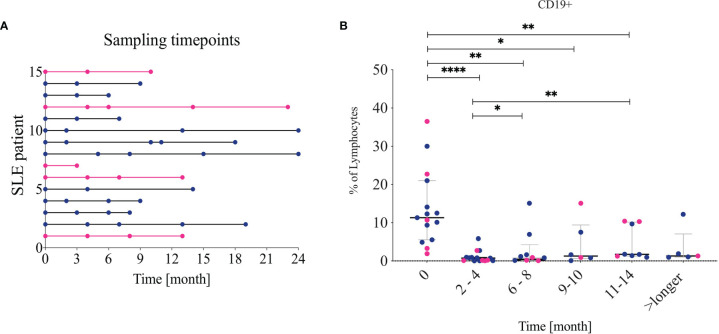
Sample timepoints and CD19 frequency **(A)** Shows which patient contributed to which timepoints. Pink lines indicate non-lupus nephritis patients. The follow-ups were combined into five different timepoints: 2-4, 6-8, 9-10, 11-14 and over 14 (longer). **(B)** Frequency of CD19+ cells from living lymphocytes over time. Lupus nephritis patients are indicated in blue while non-lupus nephritis patients are indicated in pink. * <0.05, **0.00...; *** <0.0001.

Data are presented as median and Interquartile range (IQR), if not otherwise indicated. For comparison of baseline and follow up samples, we used the Wilcoxon-matched pairs signed rank test. Differences between groups of patients were calculated using Mann-Whitney test. Patients had a different length of follow-up (**Figure 1A**). Correlation studies were done using the Spearman correlation test (non-parametric). P‐values less than 0.05 were considered statistically significant.

## Results

### Clinical Characteristics of the SLE Patients

The main clinical and demographic characteristics of the enrolled patients are displayed in **Table 1**. The patients were mostly females (86.6%), with active disease [median (IQR) SLEDAI-2K at baseline 12.0 (8.0-16.0)] and with a median disease duration of 6.1 (1.6-11.8) years.

**Table 1:** Clinical and demographic characteristics of the 15 SLE patients.

N=15		
Age [years, Median, (IQR range)]		36.7 (29.8-49.4)
Gender (n,(% Female))		13, (86.6)
Disease duration [years, Median, (IQR range)]		6.1 (1.6-11.8)
SLEDAI-2K [median, (IQR range)]		12.0 (8.0-16.0)
ANA positive [n, (%)]		15 (100)
anti-dsDNA positive [n, (%)]		9 (60.0)
Complement activation [low C3 and/or C4) (n, (%)]		8 (53.3)
Main indication for RTX (n,%)		
	Lupus nephritis	10 (66.6)
	Mucocutaneous	1 (6.6)
	CNS	2 (13.3)
	Arthritis	2 (13.3)
Concomitant cyclophoshamide [n,(%), median dose, mg (IQR)]		9 (60.0) 1000 (850-1200)
Concomitant CS pulse therapy [n,(%)]		10 (66.6)
Active oral CS treatment at baseline [n,(%),median dose, mg (IQR]		14 (93.3), 10.0 (7.5-25.0)
Antimalaria treatment at baseline [n,(%)]		7 (46.6)
Other DMARDs at baseline [n,(%)]		5 (33.3)

**Table 1**, N, number; IQR, interquartile range; SLEDAI-2K, SLE disease activity index 2000; ANA, anti-nuclear antibodies; anti-dsDNA, anti-double strand DNA antibodies; RTX, rituximab; CNS, central nervous system; CS, corticosteroids; DMARDs, disease modifying anti-rheumatic drugs.

At RTX start, several patients had serologically active disease as indicated by the prevalence of anti-dsDNA (60%) and complement consumption (53.3%). The majority of the patients received RTX because of refractory lupus nephritis (66.6%), while five were treated for other clinical manifestations (see **Table 1**). In addition, all patients but one were on oral corticosteroid treatment at baseline, at a median daily dose of 10.0 (7.5-25.0) mg, and 46.6% were on treatment with antimalarial drugs (hydroxychloroquine). Only five out of fifteen patients were receiving DMARDs at baseline: four were on mycophenolate mofetil and one on methotrexate.

Before starting RTX, the patients had been previously treated with several immune suppressants, withdrawn for either intolerance or inefficacy. Thirteen patients (86.6%) had previously received either azathioprine or mycophenolate mofetil or both, and nine patients (60%) had been previously exposed to intravenous cyclophosphamide for a median (IQR) cumulative dose of 6000 (3250–11000) mg.

### Administration of RTX and Concomitant Medications

Patients received RTX at a dose of 375 mg/m2 of BSA given weekly over four weeks (hematological schedule) in nine cases (60%), while five patients (33.3%) received a dose of 1000 mg administered two weeks apart (RA schedule), and one patient (6.7%) received a reduced dose of 500 mg administered two weeks apart.

Concomitant to RTX, intravenous cyclophosphamide was given in nine patients (60%), at a median dose of 1000 (850–1200) mg. In ten patients (66.6%), intravenous corticosteroid pulses (500 mg of 6-metylprednisolone over three consecutive days) were also administered. Eight patients (53.3%) received both associated treatments. After RTX treatment, the patients were followed with clinical evaluation every three months during the first year (or more often if clinically indicated), then on a 3-6 month basis up to 2 years depending on disease activity. Blood samples were taken in conjunction with each clinical visit (**Figure 1A**).

### Changes of the Main B-Cell Subsets After RTX Treatment

**Figure 2:**
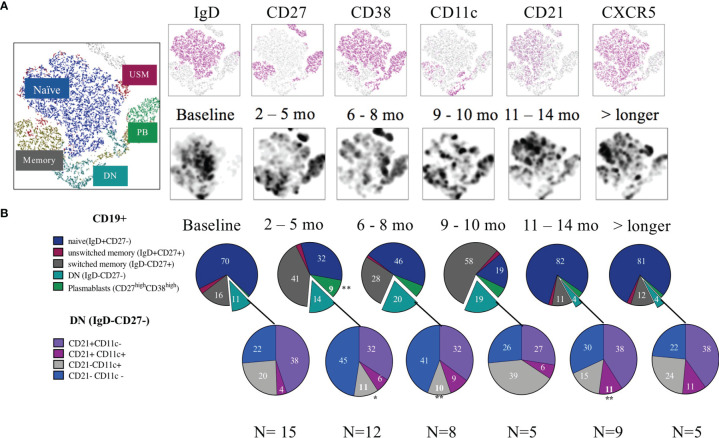
B cell subsets after rituximab treatment. **(A)** tSNE from CD19+ cells over time. The upper row shows the different markers distributed in the tSNE while the second row illustrates the density of cells in the different timelines. **(B)** Pie charts of the frequency (median) from each subset. First cells were gated for live CD19+ B cells, then for Plasmablasts (CD38++/CD27++), the rest of the cells were gated using a quadrant gate for CD27+ and IgD to identify naïve, unswitched memory, memory and double negative cells. The negative cells were further divided using the markers CD21 and CD11c. Significant change from the baseline are indicated with an *.

At baseline, SLE patients showed a median frequency of 11.3 (5.5-21.0) % CD19+ cells out of total lymphocytes. At 2–4-month follow-up after RTX treatment the CD19+ cells were significantly reduced as expected. Their median frequency diminished to 0.74% (0.67-0.94), *p<0.001* (**Figure 1B**) with three patients still having 2-5% of B cells left, 4 patients having 0.5-1% and the rest less than 0.5%. The composition of residual main B cell subsets is consistent with previous findings in the field, with plasmablasts (CD27++ CD38++) and memory B cells (IgD-CD27+) showing a significantly increased frequency, while naïve (IgD+CD27-) CD19+ cells were depleted and then normalized after approximately 9-10 months (5). The DN (IgD-CD27-) B-cell frequency increased following B-cell depletion and normalized again after approximately 11 months. The longitudinal changes observed in DN B cells, however, were not statistically significant in our cohort. The changes are illustrated in **Figures 2A, B**.

### Reduction of ABC-Like Cells After 2-4 Months of RTX Treatment

Zooming further into the DN B-cell compartment, we next investigated shifts of CD11c and CD21 expression on the DN cells. Before RTX treatment, 20.4% of DN cells expressed CD11c without concomitant CD21 expression. These IgD-CD27-CD11c+CD21- cells correspond within the memory compartment to a proxy of ABCs (herein referred to as ABC-like/CD11c+CD21-). The baseline frequencies of DN and DN/ABC-like cells did not show any correlation with the disease activity (SLEDAI-2K) in our cohort (data not shown).

Analysing the reciprocal expression of the two surface markers on DN cells, we found that 38% of the DN B cells expressed CD21 without CD11c (DN, CD11c-CD21+), 4% were double positive for CD21 and CD11c (DN, CD11c+CD21+), while 22% were double negative for CD21 and CD11c on their surface (DN, CD11c-CD21-) (**Figure 2B**).

**Figure 3:**
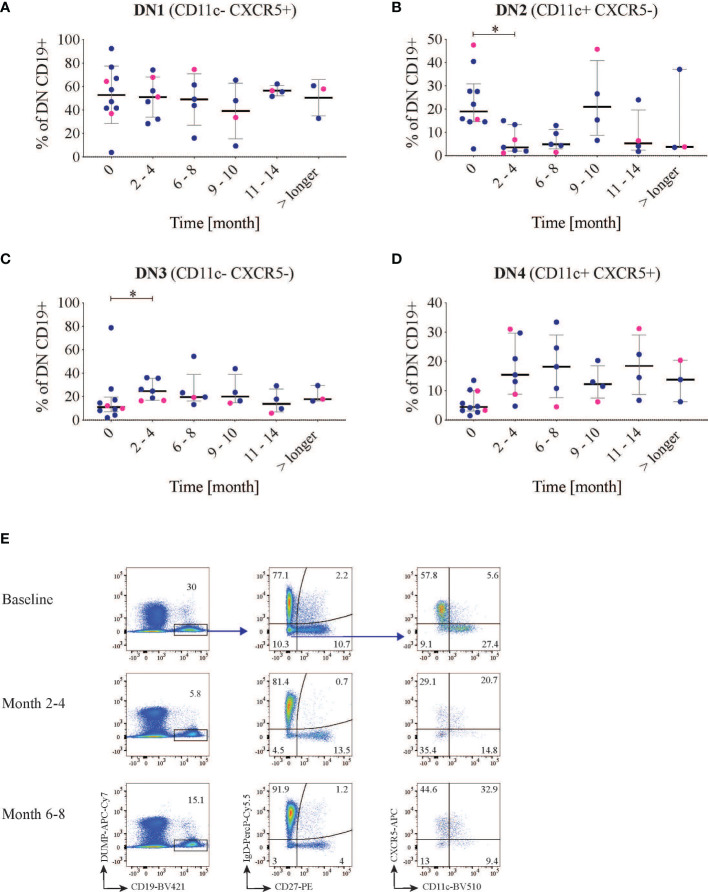
Double negative (DN) B subsets changes. CD19 B cells were first gated for IgD- CD27- cells and then divided into four different groups using the markers CXCR5 and CD11c (n=10). The graphs show the frequency of DN1 **(A)** (CXCR5+ CD11c-), DN2 **(B)** (CXCR5-CD11c+), DN3 **(C)** (CXCR5- CD11c-) and DN4 **(D)** (CXCR5+ CD11c+) B cells after rituximab treatment over time. LN patients are indicated in blue and non-LN patients in pink. **(E)** representative dot plot of the gating strategy in one patient at different time points. *<0.05.

Additionally, for 10 of the 15 patients, we also included the marker CXCR5 in the panel, which allowed us to subcategorize the DN B cells into recently defined subsets (**Figure 3**) (8). With this approach to phenotyping, DN (IgD-CD27-) memory B cells can be further divided into four sub-clusters: DN1 (CD11c-CXCR5+), DN2 (CD11c+CXCR5-), DN3 (CD11c-CXCR5-), and DN4 (CD11c+CXCR5+). In our cohort the baseline frequencies out of the DN CD19+ cells of these subclusters were 52, 19, 11 and 4.4% respectively (**Figures 3A–D**). Hence the frequency of DN CD11c+CD21- and DN2 (20.4 and 19% respectively) showed a substantial overlap.

After RTX treatment, the frequency of DN CD11c+CD21- decreased from the baseline median value of 20.4 (15.9-36.4) to 11.3 (10.4-16.3) % at 2-4 months follow-up (p=0.03). It was then maintained lower (median 10.5 (6.6-17.7), p=0.008 at 6-8 months follow-up, in order to return to values over the baseline level at the subsequent time-points. This decrease was mirrored by a parallel decrease of the subcluster DN2 (CD11c+ CXCR5-CD21-) which reduced from 19.0 (14.6-30.9) to 3.6 (2.0-13.4) % out of total CD19+ cells (p=0.02) at the first follow-up time point but repopulated after approximately 9-10 months (**Figures 2B**, **3B**). Again within the DN, IgD-CD27-CD11c-CD21- cells incremented after 2-4 months from baseline values of 22.2 (15.0-35.8) to 44.9 (32.2-65.7)% of CD19+ cells (p=0.002), and reduced again after 9-10 months (**Figure 2B**). The same applied for DN3 (CXCR5-CD11c-) cells, which increased from a median of 11.1 (7.1-19.5) to 24.7 (16.7-35.7)%, p=0.03 (**Figure 3C**), while DN4 rose but with no statically significant differences with respect to baseline (p=0.07) (**Figure 3D**). An example of cell changes in one patient is illustrated in **Figure 3E**.

Being interested in possible clinical correlations, we explored whether the early decrease in DN CD11c+CD21- cells showed association to clinical response. Overall, in our cohort the SLEDAI-2K score decreased 6.0 (2.0-10.0) points (Δ-SLEDAI-2K) from baseline to 6 months follow-up. With reservation for the small samples size, we found no association between clinical response at 6 months (defined as decrease in total SLEDAI-2K≥4) and reduction in DN CD11c+CD21- cell frequency, nor any correlation with SLEDAI-2K values at six months.

In contrast, when only looking at the CD11c+CD21- population irrespective of their IgD and CD27 status, i.e. not confined to DN memory cells, there was no significant reduction after 2-4 months, although we observed a significant increment in CD11c-CD21- and CD11c+CD21+ CD19+ cells after RTX treatment (**Supplementary Figures S2C, D**). The CD19+ B cells expressing CD21+ (but negative for CD11c) did instead decrease (**Supplementary Figure S2B**). This reduction was expected, since part of the circulating CD21+ cells are de facto naïve B-cells, which are most typically susceptible to B-cell depletion with RTX.

Overall, we could see a significant reduction of DN2/ABC-like after 2-4 months from RTX treatment while CD19+CD11c-CD21- and DN3 cells increased.

### T-Cell Subsets Frequencies After RTX Treatment

**Figure 4:**
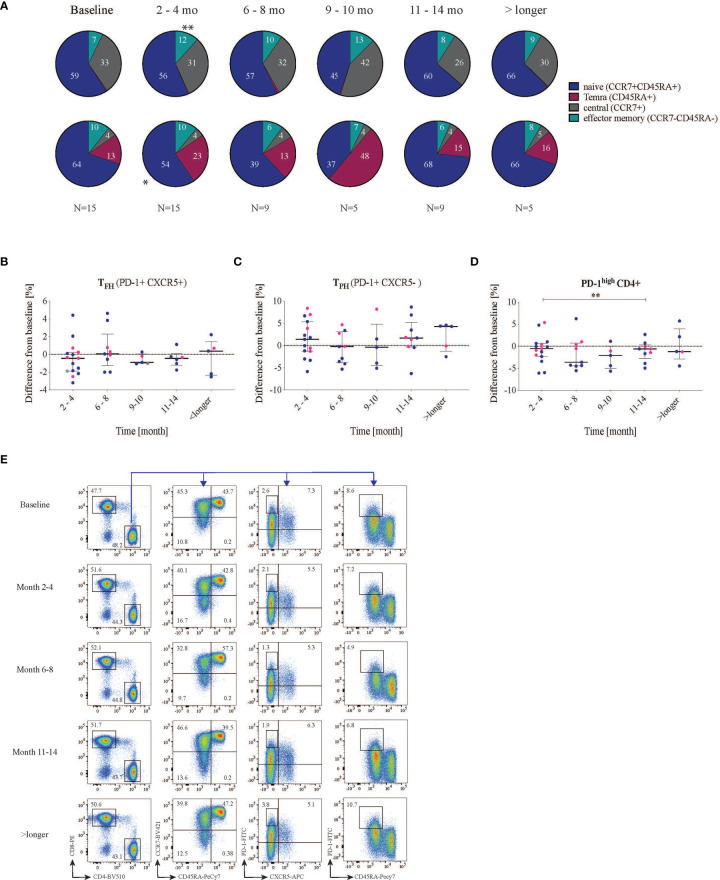
Effect on T cell subsets after RTX treatment. Cells were first gated for living CD3 and then divided into CD4 and CD8 cells. **(A)** CCR7 and CD45RA were used to subdivide the T cell subsets into naïve (CCR7+ CD45RA+), central memory (CCR7+ CD45RA-) and effector memory (CCR7- CD45RA-) as well as T_EMRA_ (CCR7-CD45RA+) of both CD4 and CD8 cells. **(B–D)** Shows the difference in frequency from baseline (- values indicate reduction while + values indicate increase from baseline) **(B)** T follicular helper cells (PD-1+CXCR5+) did not change significantly in frequency. **(C)** PD-1+ CXCR5- T cells divide into two groups, one with reduced PD-1 frequency and one with increased PD-1 frequency. **(D)** PD-1^high^ CD4+ cells decrease after 2-4 months and significant reduction can be seen between the months 2-4 and 11-14. Lupus nephritis patients are displayed in blue while non-lupus nephritis patients are shown with pink dots. **(E)** representative dot plot of the gating strategy in one patient at different time points. * <0.05, **0.00.

Since autoantibody production plays an important role in SLE, we further wanted to investigate T-cell phenotypes associated with B-cell help. We also included markers to enable global analyses of both CD4+ and CD8+ T cells. Hence, we first analyzed general T-cell phenotypes using the markers CD45RA and CCR7 to identify effector memory, central memory, T_EMRA_ and naïve T cells. After RTX treatment, the proportion of effector memory T cells (median of differences for CD4: 3.2%, p=0.0006; for CD8: 1.6% p=0.04) and T_EMRA_ (median of differences CD4: 0.1%, p=0.001; CD8: 3.6% p=0.03) increased in both CD4 and CD8 T-cell subsets, while the proportion of naïve T cells decreased (median of differences CD4:- 2.1%, p=0.2; CD8: -8.2% p=0.01) (**Figure 4A** and **Supplementary Figure 3**). Central memory T cells which historically have been attributed as key for T cell help to B cells (29) seem not to be affected by the treatment.

Subsequently, for a more granular view, we focused on T_FH_ (PD-1+CXCR5+) cells due to their direct implication for B-cell help and autoantibody production. Before treatment, the SLE patients exhibited a median frequency of T_FH_ of 3.6 (1.5-5.9)%, with LN patients having a higher frequency of circulating T_FH_ (4.63 (2.6-6.0) % compared to non-LN patients (1.5 (0.4-5.6)%), however without reaching statistical significance (p=0.09, data not shown). Following RTX treatment, the proportion of circulating T_FH_ (CXCR5+ PD-1+) among CD4+ T cells showed a non-significant median reduction from (3.6 (1.5-5.9)% to 2.4 (1.2-5.5)%, p=0.2) at 2-4 months and stayed the same at the subsequent time points (**Figure 4B** and **Supplementary Figure 4A**).

Next, we were interested in the general expression of PD-1 on CXCR5- CD4+ T cells [which include the recently described subset of peripheral helper T cells (T_PH_)]. PD-1 is an inhibitory receptor mainly expressed on activated, effector and so-called exhausted T cells (30). We observed that PD-1+CXCR5- CD4+ T cells cluster into two groups of patients with apparent low and high frequencies, however, with no apparent difference between patient subsets (data not shown). When looking at changes in this subset with respect to baseline frequencies, we did not observe significant changes over time (**Figure 4C** and **Supplementary Figure 4B**). Elevated levels of PD-1^high^ CD4+ T cells have been described in SLE patients and to correlate with disease severity (31). We therefore analyzed cells expressing high levels of surface PD-1 (PD-1^high^ CD4+). In our cohort, at baseline, PD-1^high^ CD4+ T cell frequencies were higher in LN patients compared with non-LN patients, with a trend towards a statistically significant difference (6.1 (4.0-8.6) vs 3.0 (1.5-5.3) % out of CD4+, p=0.055 (data not shown). The SLEDAI at baseline did not correlate with the frequency of PD-1^high^ T cells (r=0.03, p=0.9, **Supplementary Figure 4D**). Interestingly, 2-4 months after RTX treatment, in most patients the frequency of PD-1^high^ cells either reduced or stayed the same (median reduction from baseline= -0.53% (-2.3-+0.62), p=0.28) (**Figure 4D**). After 11-15 months of RTX treatment, the frequency of PD-1^high^ CD4+ T showed a median reduction from baseline of -0.62% (-3.0-+0.31) which was statistically significant with respect to the reduction at 2-4 months (p=0.04, **Figure 4D**, and **Supplementary Figure 4C**). An example of the gating strategy is provided in **Figure 4E**.

To summarize, T_EMRA_ and T effector memory cells frequencies increased after 2-4 months of RTX treatment. The frequency of circulating T_FH_ cells in peripheral blood did not change significantly. Seemingly, no significant changes were observed in PD-1+CXCR5- CD4 T cells, while PD-1^high^ CD4+ cells frequency showed significant fluctuation between intermediate time-points.

### Comparative Analysis of the B-and T-Cell Autoimmunity Associated Phenotypes in Patients DevelopingRTX-Related Immunogenicity

The 15 patients analyzed in this study were also included in a recently published paper investigating the prevalence of anti-drug antibodies (ADA) in our SLE cohort (28). In the mentioned paper, ADA positive SLE individuals showed higher B-cell counts at about six months follow-up with respect to ADA negative individuals. Since the patients here constitute a subgroup of the mentioned study, we wanted to look whether any relation between ADA and the cell phenotypes of interest may emerge. Of the 15 patients, 8 were ADA positive at the 6-8 months follow-up timepoint. All but one had received RTX for LN.

The observation of higher CD19+ frequencies at follow-up was here partly confirmed by a trend towards higher median values in ADA positive individuals (p=0.06, data not shown).

Moreover, patients later exhibiting ADA, had higher median levels of naïve B cells at 2-4 months, although the difference towards ADA negative patients did not show statistical significance (data not shown). When exploring the B-cell subsets of interest, we did not observe differences in the frequencies of CD11c+CD21- cells at any timepoint.

Still, looking at the larger DN B-cell subset, we observed that, at 2-4 months follow-up, ADA negative patients had significantly higher median DN frequencies compared to ADA positive patients (29.9 (18.4-38.2)% vs 11.4.0 (7.0-14.1)%, *p=0.02*, **Supplementary Figures 5A, B**). Such difference could not be confirmed at the same timepoint for the DN CD11c+CD21- cells, nor when these cells were stained also for CXCR5 lack of expression (data not shown).

ADA positive patients, at the same timepoint of 2-4 months from RTX start, showed a more pronounced plasmablast expansion with respect to baseline (Wilcoxon test *p=0.01*). Instead, the rise of plasmablast frequencies in ADA negative patients was not significant in the longitudinal analysis (**Supplementary Figure 5C**).

No differences in the frequencies of T_FH_ could be detected. However, when focusing on the PD-1^high^ CD4+ cells we could observe a tendency towards a higher frequency in ADA positive individuals at the timepoint 2-4 months and 11-14 months (p=0.07 and 0.06 respectively) (**Supplementary Figure 5D**).

## Discussion

Our study explored the effect of RTX on lymphocyte subsets associated with autoimmunity. We focused on ABC-like memory B-cells, T_FH_, T_PH_ and PD-1^high^ CD4+ T cells in 10 lupus nephritis and 5 non-lupus nephritis SLE patients. In the early follow-up, we observed a significant reduction of DN2/ABC-like frequency, while CD11c-CD21-/DN3 cells frequency increased. The frequency of T_FH_ cells was not significantly influenced by the treatment, but shifts in the frequency of PD-1^high^ CD4+ T cells were observed between early and later follow-up.

The interest in these subsets was driven by the emerging data supporting their direct involvement in autoimmunity over the last decade. Today, large data sets have explored the phenotypical and functional characteristics of both B and T cells centrally involved in autoimmunity, albeit some of the details are still partly debated. Memory B cells have been shown as yielding a rather high degree of complexity and heterogeneity, and several distinct subsets of atypical memory B-cells have been described (7). Among these the DN cells and within them DN2 and ABC-like, which are substantially overlapping. Collectively, these different phenotypes share the common feature of developing in the context of antigenic stimulation, and possibly represent stages of post-activation. Indeed, they share features of emergence from GC reactions. However, they do not seem to be cells that accumulate over time and serve as innocent bystanders of pathogenic events; rather, they are possible actors in the complex interplay that leads to the generation of plasmablasts and plasma cells (7). Such events are largely dependent on interactions with T cells, either in typical follicular or extra-follicular responses. T cells able to provide help to B cells and guide them towards the final steps of differentiation have been shown crucial in autoimmune diseases and especially in SLE, and accumulating evidence suggest a role of T-cells in the generation of atypical memory subsets (19, 21, 32). Therefore, understanding the impact of currently used therapeutics on these cellular phenotypes is of high relevance for optimizing their use and elaborate future strategies and approaches.

As previously reported, naïve B cells are sensitive to BCD leading to their decrease and a relative expansion of memory and plasmablasts at early follow-up (22). DN B-cells were first identified while studying the effects of RTX in SLE patients. Afterwards, they were characterized as memory cells and shown to be expanded in SLE and correlate with disease activity, in studies not directly focusing on RTX treated SLE patients (5, 6). In later years, the DN subset was further investigated, and the DN2 phenotype (CD11c+CD21-CXCR5-) emerged as the predominant form of DN in SLE patients. Once again, association with disease activity was confirmed. DN2 cells express the transcription factor T-bet, exhibit features of extrafollicular differentiation in inflamed tissues, and are able to quickly differentiate into antibody producing cells (8).

In our study, we investigated DN subsets in the context of BCD. At baseline, we found overlapping median frequencies in ABC-like DN and DN2, with values not diverging from what was observed in previous studies, performed in SLE but not in the context of BCD (8). In the early follow-up, hence in a status of BCD, these phenotypes were significantly reduced in frequency. In the residual B-cell pool, we observed an increase of the recently defined DN3 phenotype. This phenotype emerged in a detailed phenotypical analysis which compared B-cell subsets in severe COVID-19 patients and SLE patients underscoring extrafollicular responses in both SLE and severe COVID-19. Moreover, it was further confirmed that DN3 expansion is a characteristic of severe COVID-19, and reaches the highest proportion in critically ill patients (33, 34).

DN1 have been shown to be the prevalent DN phenotype in healthy individuals and are transcriptionally related to switched memory B cells, while DN2 and DN3 have been found expanded mostly in pathologic conditions. DN3 cells are partially overlapping with DN1, and initial analysis has suggested they may represent subsets originating by diverging differentiation pathways. DN1 and DN3 cells resemble early memory precursors (8, 33, 35).

In a recent paper, DN cells first defined phenotypically with a classical flow cytometry approach, were further dissected using a single-cell transcriptomic approach followed by a RNA trajectory analysis. With this approach, DN cells appeared a heterogeneous population in which DN3 precedes as stage DN2 and both belong to a separate developmental pathway-originating from naïve cells-compared to DN1 and DN4 (here not defined though based on CD11 and CXCR5 expression as in our study). According to this study, DN1 show features of activated B-cells and, with DN4 seem to represent an evolutionary pathway close to classical memory cells. On the contrary, DN2 appear as the terminal stage of differentiation of a divergent and distinct B-memory evolutionary pathway, T-bet driven and more resembling atypical memory (35).

The cells that we, in our study, characterize as DN4, may possibly overlap with the population defined on the basis of RNA expression. We did not see significant dynamic changes in our cohort for what concerns this CD11c+CXCR5+ subset, although a trend towards a significant increase at early follow-up could be detected. What is interesting, in this yet not well defined subset, is its enrichment for IgE (35). This feature might be of interest in light of the potential immunogenicity of RTX, and provide a translational link for better characterizing the role of IgE ADA for example in the clinical consequences of ADA formation.

Interestingly, the total frequency of CD11c+CD21-cells did not change after treatment, highlighting the diversity of CD11c+ cells and the need for multiparameter approaches when studying these cells.

The proportions of T_EMRA_ and effector memory cells amongst CD4+ and CD8+ T cell frequencies increased slightly after RTX treatment. We did not see a significant change in the frequency of circulating CXCR5+ T_FH_ cells after RTX treatment. However, we did not study different subtypes of T_FH_ cells. Human circulating T_FH_ are suggested to consist of three major functionally distinct subsets: T_FH_1, T_FH_2 and T_FH_17 and are sub-defined by CXCR3 and CCR6 expression. Moreover, an imbalance of circulating T_FH_, towards T_FH_2 cells has been reported in SLE, and to correlate with an increase of DN B cells (36).

An increase of PD-1^high^ CXCR5- CD4 T cells has been found in peripheral blood of active SLE patients (31, 37). PD-1^high^ CD4+ T cells have been associated with relapses and to correlate with disease activity (31). In our study, the frequency of PD-1^high^ cells did not correlate with SLEDAI at baseline, however the sample size of our study could influence the finding. We observed though a tendency towards higher frequencies of PD-1^high^ CD4+ cells in LN patients with respect to non-LN patients. This finding yields relevance and might be replicated in larger studies. PD-1^high^ CD4+ T cells transiently decreased upon RTX therapy during later follow-up, although the significance of these changes remains elusive, given the limited inference allowed by our small cohort; therefore we could not replicate previous observations concerning shifts in T_PH_ cells according to different degrees of disease activity (38).

A small proportion of T cells has been demonstrated to express CD20 (39), and would thus be potentially affected by RTX treatment. Since we did not include CD20 in our panel, we cannot assess whether the additional deletion of these cells might have influenced our results. Moreover, the main limitations of this study can be identified in the small sample size, the heterogenous nature of SLE together with the exposure to previous treatments, which could have influenced the baseline proportions of the lymphocytes subsets, and account for the heterogeneity of the cell distributions at baseline. Also, the concomitant administration of corticosteroids and cyclophosphamide may have contributed to some changes in the peripheral blood distribution of the cellular subsets. Although not ideal for studying how the different cell phenotypes are influenced by RTX, such exposures reflect the real life setting in which the study was conceived and performed. Another potential limitation is the lack of measuring of the absolute number of cells, only allowing observations of changes in the proportions between subsets. This aspect warrants further replication of the findings.

However, our study shows preliminary exploratory findings on the possible effects of RTX on ABC-like memory cells, which are a B cell subset of increasing interest for its implications in autoimmunity. Future efforts in pharmacological developments might point at selectively targeting those cell subsets mostly enriched in autoreactivity while sparing non-autoreactive clones.

With the consideration of the limitations of the sample size, and the possibility that relevant differences may not emerge, the inclusion of RTX-immunogenicity data as assessed by presence of ADA provides some preliminary although interesting insights into the possible biological origin of the ADA. It has to be considered that ADA develop during B-cell depletion towards a new antigen provided to the immune system *via* the treatment. Since RTX causes a rapid decline of the naïve B cells, although pre-existing natural immunity or cross-reactivity cannot be excluded, we must consider that the cells responding to RTX and giving rise to ADA formation must be located in the residual B cells. Therefore, if not originating from the canonical naïve B cells, they may arise from other cell compartments less susceptible to RTX action. One of such compartments may be the DN cells, and the lower frequencies we observed in ADA positive patients, coupled with a more pronounced rise in plasmablasts, may indicate a recruitment of plasmablast within the DN compartment. In this study we observed a difference (although not statistically significant) in median levels of naïve residual B cells, which suggests a primary resistance to BCD as an alternative or complementary mechanism in those patients later developing ADA. Whether the studied patients were already ADA positive at 2-4 months follow-up could not be ascertained, given the possible interference of residual circulating drug on the ADA assay. Also, ADA positive patients at six months had lower levels of DN cells at 2-4 months, and showed a more significant increase of plasmablasts, which suggests different degrees of imbalances in the residual B-cell pool implicated in immunogenicity. This observation may have implications beyond the explanation of RTX immunogenicity, and be relevant in the context of autoimmunity itself as well as for the exposure to other foreign antigens (infections, vaccines). The role of PD-1 CD4+ in this context may also point towards the direction of promoting ADA development providing B-cell help to B-cells evolving into ADA forming cells.

In conclusion, the importance of T-B cell interactions in SLE pathogenesis is strongly supported by altered frequencies in specific lymphocytes subsets, ABC-like and T_FH_/T_PH_ respectively. Here, in the context of RTX treated SLE we could detect a clear reduction of the proportions of ABC-like and less clear alterations in PD-1^high^ T cells after treatment with RTX.

Our data further suggests that anti-CD20 mediated B cell depletion affects not only the frequency of specific B-cell subsets but also influence CD4+ T-cell subsets that are clinically relevant.

In this context we also observed alterations in the B- and T- cells which may have implications for RTX directed immunogenicity, a preliminary observation that deserves further research.

Further insight into the effects of B-cell depletive agents and other SLE treatments on B- and T- cell subsets involved in critical steps of SLE immune-pathogenesis should be pursued.

## Data Availability Statement

Original raw data will be provided by the corresponding and the senior author of this paper upon reasonable request.

## Ethics Statement

The studies involving human participants were reviewed and approved by Regional Ethics Committe Stockholm (Regionala Etikprövningsnämden). The patients/participants provided their written informed consent to participate in this study.

## Author Contributions

FF, VM, NS, and IG conceived and planned the study. FF, NS, and VM wrote the manuscript. NS, RS, and KC generated the flow-cytometry data. NS and FF analyzed the flow cytometry data. ND and AF-H generated the data on anti-drug antibodies, which were analyzed and interpreted by ND and FF. All authors read and critically revised the manuscript.

## Funding

The study was financed by Region Stockholm ALF (Avtal för Läkarutbildning och Forskning), The Swedish Rheumatism Association, King Gustaf V’s 80-year Foundation, Ingegerd Johansson’s Fund, Karolinska Institutet Foundations, and the Swedish Research Council (Vetenskapsrådet).

## Conflict of Interest

The authors declare that the research was conducted in the absence of any commercial or financial relationships that could be construed as a potential conflict of interest.

## Publisher’s Note

All claims expressed in this article are solely those of the authors and do not necessarily represent those of their affiliated organizations, or those of the publisher, the editors and the reviewers. Any product that may be evaluated in this article, or claim that may be made by its manufacturer, is not guaranteed or endorsed by the publisher.
