# Supplementary figures and images for: Rituximab in Systemic Lupus Erythematosus: Transient Effects on Autoimmunity Associated Lymphocyte Phenotypes and Implications for Immunogenicity
Source: Front Immunol. 2022 Apr 8;13:826152. doi: 10.3389/fimmu.2022.826152 (PMC9027571; doi:10.3389/fimmu.2022.826152)

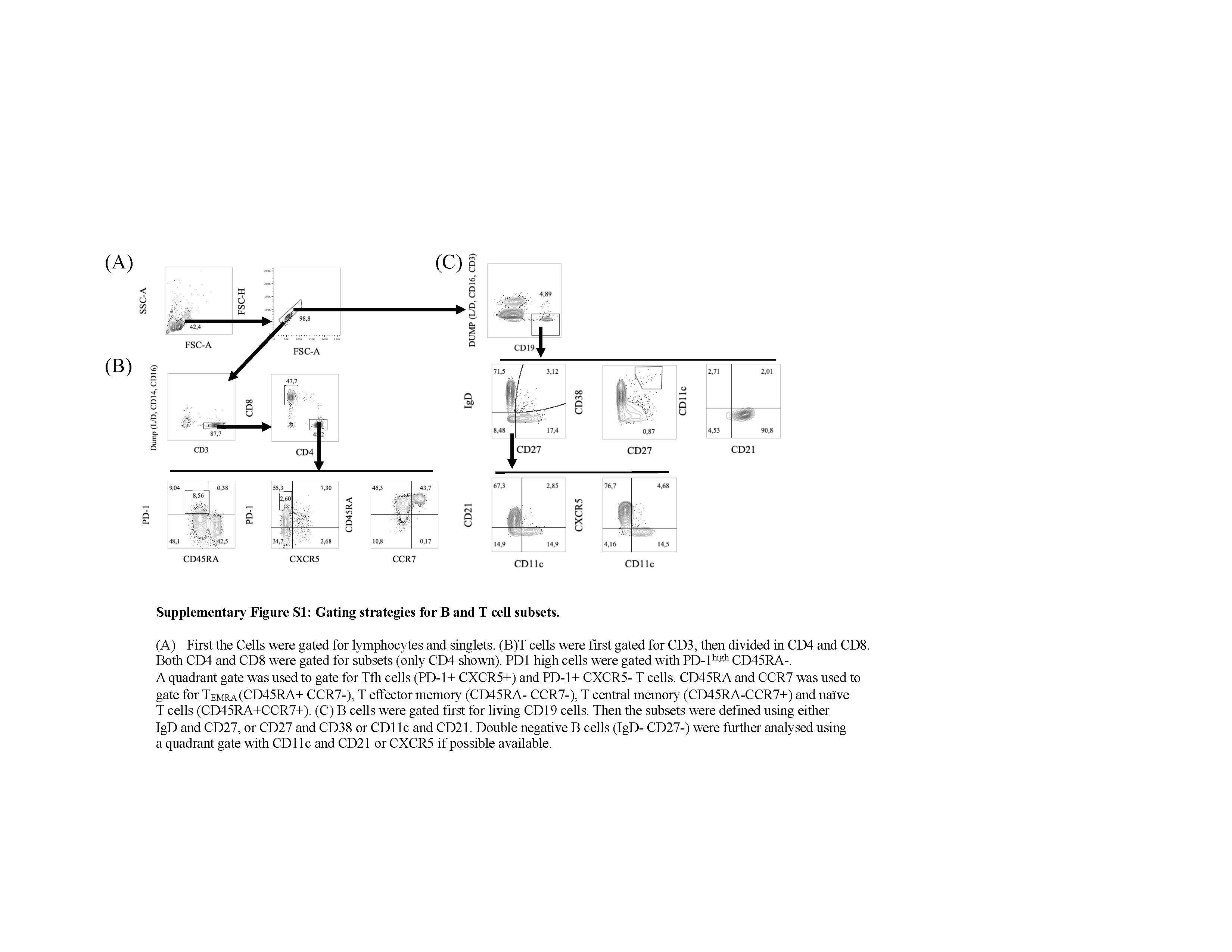

Supplement: Supplementary file 1 [file Image_1.jpeg]

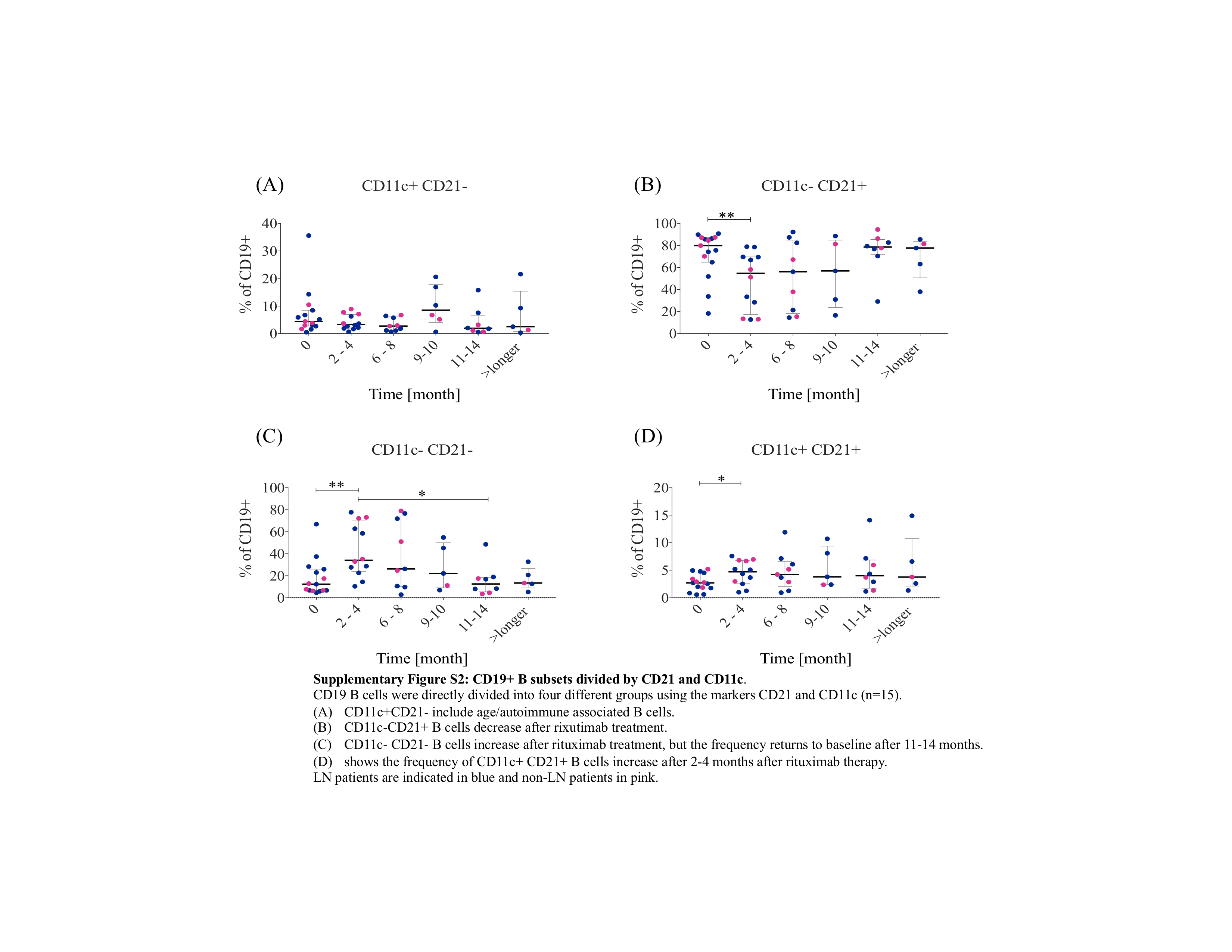

Supplement: Supplementary file 2 [file Image_2.jpg]

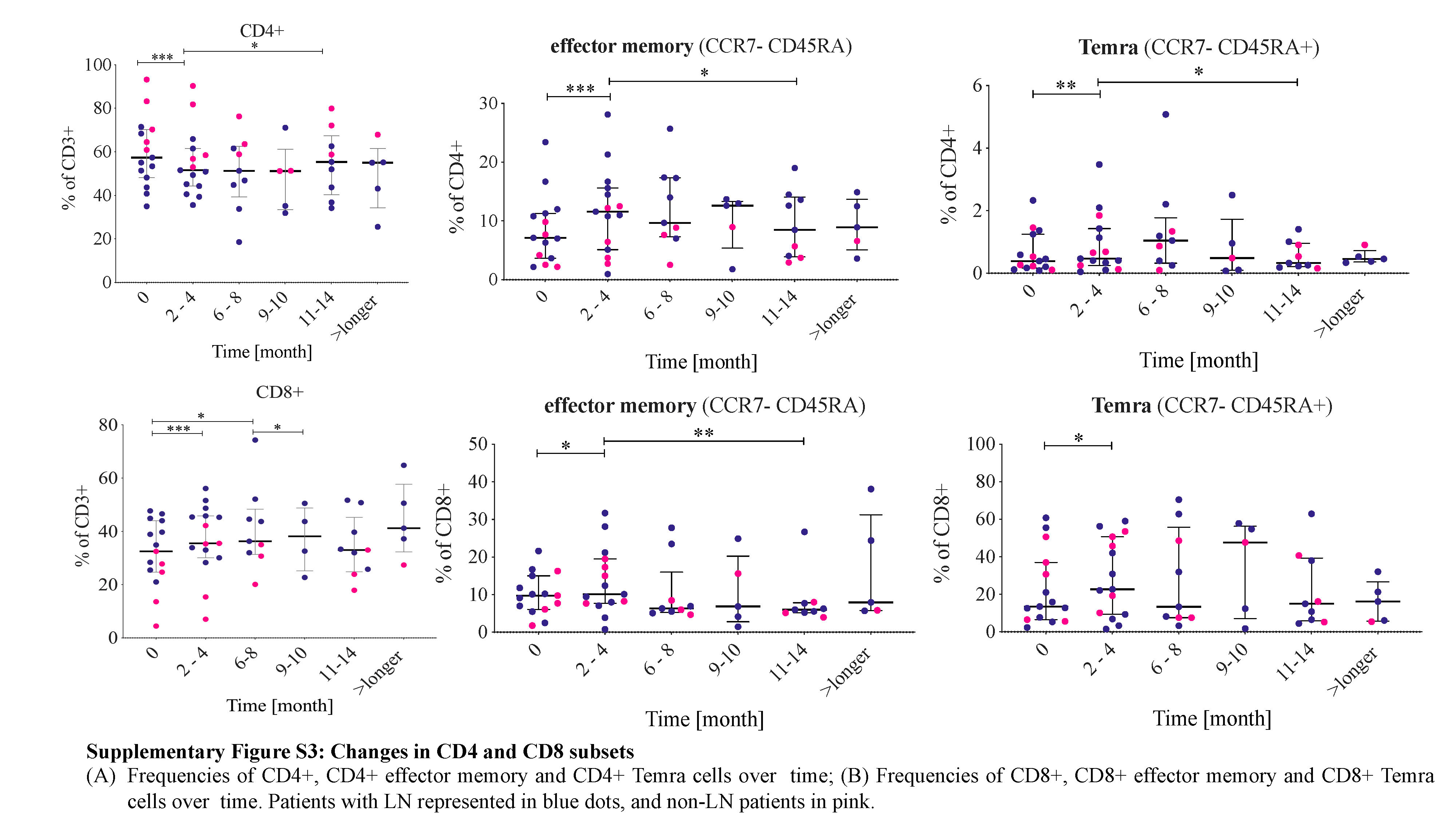

Supplement: Supplementary file 3 [file Image_3.jpg]

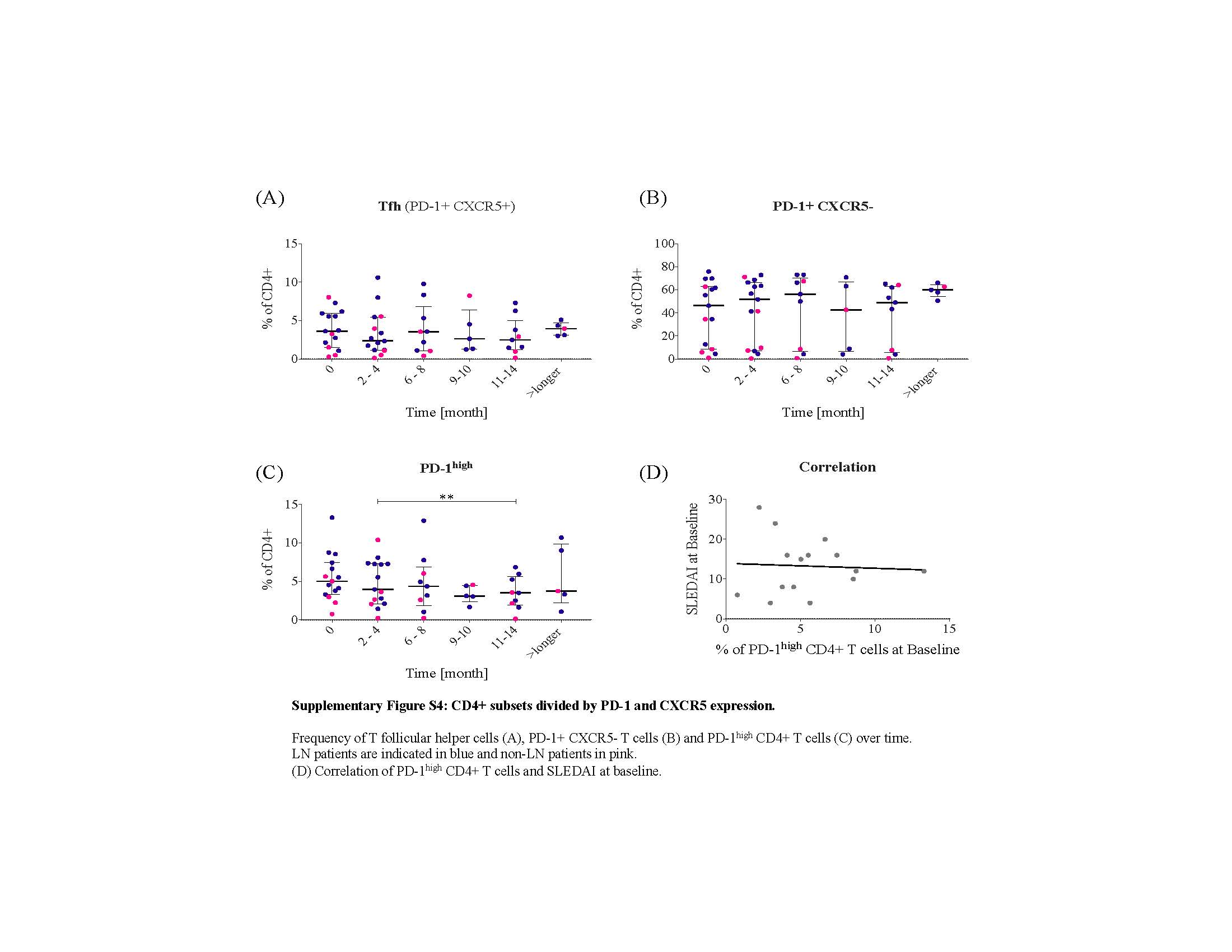

Supplement: Supplementary file 4 [file Image_4.jpeg]

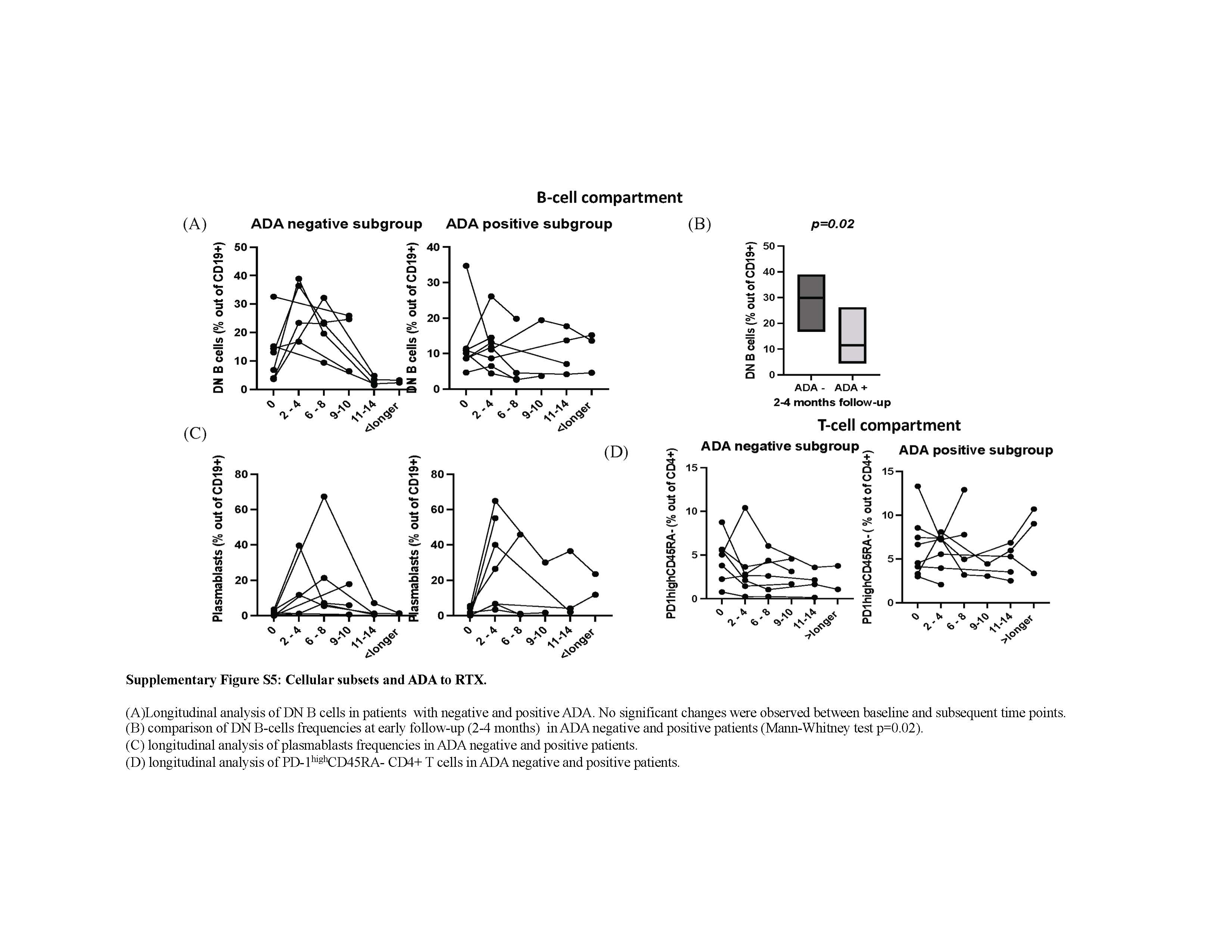

Supplement: Supplementary file 5 [file Image_5.jpeg]
