# Supplementary material for: Rituximab in Systemic Lupus Erythematosus: Transient Effects on Autoimmunity Associated Lymphocyte Phenotypes and Implications for Immunogenicity
Source: Front Immunol. 2022 Apr 8;13:826152. doi: 10.3389/fimmu.2022.826152 (PMC9027571; doi:10.3389/fimmu.2022.826152)
Supplement: Supplementary file 6 [file Table_1.docx]

Supplementary Material

***Supplementary Table S1*: Flow Cytometry Marker for B and T cells**

| **Flurochrome** | **Marker** | **Clone** | **Company** |
| --- | --- | --- | --- |
| Near-IR | LIVE/DEAD™Dead Cell Stain Kit | | Invitrogen |
| APC-H7/Per-CpCy5.5 | CD3 | UCHT1 | BD Biosciences |
| BV510 | CD4 | SK3 | BD Biosciences |
| PE | CD8 | RPA-T8 | BD Biosciences |
| BB515 | PD-1 | EH12.1 | BD Biosciences |
| APC | CXCR5 | J252D4 | BD Biosciences |
| PE-Cy7 | CD45RA | HI100 | BD Biosciences |
| BV421 | CCR7 | 150503 | BD Biosiences |
| PerCp-Cy.5.5 | IgD | Ia6-2 | BD Biosciences |
| PE | CD27 | L128 | BD Biosciences |
| PE-Cy7 | CD38 | HIT2 | BD Biosciences |
| BV421/ APC-H7 | CD19 | HIB19 | BD Biosciences |
| FITC | CD21 | B-ly4 | BD Biosciences |
| BV510 | CD11c | B-ly6 | BD Biosciences |
| APC-H7/ BV510 | CD16 | 3G8 | BD Biosciences |
| APC-H7/ FITC | CD14 | MφP9 | BD Biosciences |
